# A cohort study on anxiety and perceived recovery 3 and 12 months after mild to moderate stroke

**DOI:** 10.3389/fneur.2023.1273864

**Published:** 2023-10-10

**Authors:** Linda Nelsone, Lena Rafsten, Tamar Abzhandadze, Katharina S. Sunnerhagen

**Affiliations:** ^1^Faculty of Residency, Riga Stradins University, Riga, Latvia; ^2^Riga East University Hospital, Riga, Latvia; ^3^Department of Clinical Neuroscience and Rehabilitation Medicine, Institute of Neuroscience and Physiology, Sahlgrenska Academy, University of Gothenburg, Gothenburg, Sweden; ^4^Department of Occupational Therapy and Physiotherapy, Sahlgrenska University Hospital, Gothenburg, Sweden; ^5^Rehabilitation Medicine, Neurocare, Sahlgrenska University Hospital, Gothenburg, Sweden

**Keywords:** cerebrovascular accident, stroke, anxiety, Stroke Impact Scale, perception, longitudinal study, functional recovery, Hospital Anxiety and Depression Scale

## Abstract

**Background:**

Anxiety is a common complication of stroke, affecting one in every three stroke survivors. Stroke recovery is a dynamic process, with most recovery occurring within the first 3 months. However, how anxiety affects this dynamic remains unknown. Therefore, this study aimed to investigate how anxiety affects perceived recovery at 3 and 12 months after stroke. Additionally we also examined the change in perceived stroke recovery from 3 to 12 months and its relationship with anxiety.

**Methods:**

In this longitudinal study patients with stroke were enrolled at Sahlgrenska University Hospital, Gothenburg, Sweden. The Hospital Anxiety and Depression Scale was used to assess anxiety, and the Stroke Impact Scale was used to assess perceived recovery 3 and 12 months after the stroke. The difference in perceived stroke recovery between the anxiety and no-anxiety groups at 3 and 12 months was analyzed. Changes in perceived stroke recovery were calculated and trichotomized from 3 to 12 months based on clinically significant positive changes (+10 points or more), clinically important negative changes (−10 points or less), or no changes (±9). At 3 and 12 months after the stroke, negative and positive recovery was compared to no change in recovery regarding anxiety scores.

**Results:**

This study included 99 patients (44.4% female, median age, 77 years). At 3 and 12 months after the stroke, the median recovery score was 80 out of 100. At 3- and 12-months 17.6 and 15.7% of the patients experienced anxiety, respectively. At both time points, there was a significant association between anxiety and lower perceived stroke recovery (at 3 months: *p* < 0.001; and 12 months *p* = 0.002). Among participants with anxiety at 3 or 12 months after stroke, a positive change in recovery from 3 to 12 months was identified (3 months, *p* = 0.004 and 12 months, *p* = 0.0014).

**Conclusion:**

Anxiety symptoms following a stroke are associated with lower levels of perceived stroke recovery for at least 1 year after the stroke. Identifying patients with anxiety early after stroke may be beneficial for identifying those at risk of lower recovery.

**Clinical trial registration:**ClinicalTrials.gov, identifier [NCT01622205]. Registered on June 19, 2012 (retrospectively registered).

## Introduction

Stroke is a sudden neurological deficit caused by acute cerebrovascular, ischemic, or hemorrhagic focal damage (1). In 2019, the incidence of stroke was 12 million, with a global prevalence of 101 million (2), making it the third leading cause of disability and death worldwide (2). More people survive strokes, increasing the likelihood of living with long-term physical, cognitive, and psychological consequences (2, 3).

Psychological consequences of stroke can contribute to poor outcomes (4–7). Anxiety is the second most common neuropsychiatric consequence of stroke (8), affecting approximately 20–30% of stroke survivors at some point after the stroke (9, 10). According to the World Health Organization, approximately 38% of the general population experience some form of anxiety (11). A longitudinal study concluded that anxiety levels did not change significantly during the first year after stroke (12). Anxiety can be defined as a feeling of worry, uneasiness, fear, or dread (13). Anxiety after a stroke is associated with a reduced quality of life, increased disability, depression, and dependency on activities of daily living (7, 10, 14–17).

Other common stroke consequences include impaired motor and sensory functions, cognitive function, participation restrictions, and daily activity limitations (18). Patients with stroke-related impairments undergo the most intensive recovery during the first 3 months and continue for at least 1 year after the stroke (19). However, patients perceive their improvement differently, and all stroke consequences can be related to how they perceive their recovery. Longitudinal changes in perceived recovery have been investigated, and significant improvements have been found between 3 and 12 months after stroke (20). Perceived recovery can be defined as the subjective perception of a patient’s physical and psychological improvement (21). The Stroke Impact Scale (SIS) is a common used tool to assess how patients with stroke perceive recovery. It was developed to assess the perceptions of patients on the impact of stroke on dimensions such as emotion, communication, memory, thinking, social role, and perceived global stroke recovery (22).

Individually, studies have been conducted on post-stroke anxiety and perceived stroke recovery. Anxiety is a common condition following stroke (9, 10) and is associated with a reduced quality of life (23). Furthermore, the tool of perceived stroke recovery is used to assess the subjective thoughts of patients about their recovery process (21). However, the role of anxiety in stroke recovery is unknown, necessitating further research in this field.

Hence, this study aimed to investigate the association between anxiety and perceived recovery at 3 and 12 months after stroke. Furthermore, this study aimed to investigate the change in perceived stroke recovery from 3 to 12 months and its relationship with anxiety.

## Materials and methods

### Study design and sample

The data in this longitudinal study was based on data from the Gothenburg Very Early Supported Discharge study (GOTVED) (24), which is a randomized controlled trial with blinded assessors. Patients admitted to the stroke unit of Sahlgrenska University Hospital, Gothenburg, Sweden, were enrolled between 2011 and 2016. Sahlgrenska University Hospital is the largest hospital network in Sweden, providing emergency and basic care to 700,000 inhabitants in the Gothenburg region and highly specialized care to 1.7 million inhabitants in Region Västra Götaland. Moreover, the hospital is responsible for thrombectomy treatment in the region (24).

Participants were included in this study if they had a stroke diagnosis (ischemic or hemorrhagic) according to the World Health Organization criteria (25), were ≥ 18 years old at the time of stroke onset, and had data on anxiety and perceived stroke recovery between 3 and/or 12 months after stroke. The exclusion criteria were a National Institutes of Health Stoke Scale (NIHSS) score of >16 and a Barthel Index (BI) score of <50. Additionally, patients with a life expectancy of <1 year (e.g., those with severe malignancy) or who were unable to speak or communicate in Swedish prior to stroke were excluded. Additional information regarding the inclusion and exclusion criteria for the GOTVED trial can be found in a previous study (24).

### Ethics approval

This study was approved by the Swedish Ethical Review Authority (formerly Regional Ethical Review Board) in Gothenburg (registration numbers: 426-05 and 042-11), and was conducted in accordance with the Declaration of Helsinki. All participants provided written informed consent before the longitudinal study.

### Procedure

**Figure 1:**
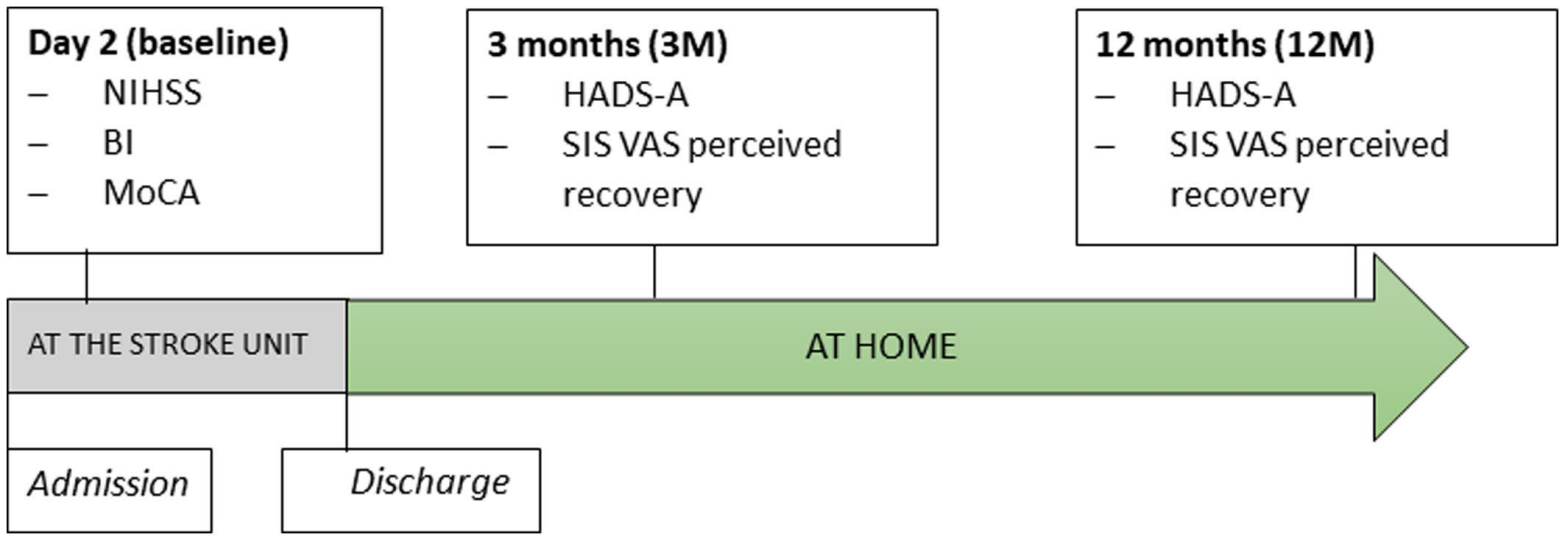
The timeline of assessments. NIHSS, National Institute of Health Stroke Scale; BI, Barthel Index; MoCA, Montreal Cognitive Assessment; HADS-A, Hospital Anxiety and Depression Scale – Subscale of Anxiety, SIS, Stroke Impact Scale; VAS, Visual Analog Scale of perceived recovery.

An experienced nurse assessed the neurological deficits of stroke survivors 36–48 h after admission to the stroke unit (referred to as baseline). Occupational therapists assessed basic activities of daily living (ADL) performance and screened for cognitive function. A trained, blinded researcher who was not affiliated with the stroke unit collected follow-up data between 3 and 12 months after stroke. Following discharge, all assessments were conducted at the home of the participants. Stroke survivors completed self-report questionnaires under the supervision of the assessor to report their anxiety and perceived stroke recovery. The assessor could assist the participant when something was unclear or required additional explanation (24). Figure 1 shows detailed information on the assessment time points and instruments.

### Study variables

The SIS version 3.0 was used to assess global perceived stroke recovery 3 and 12 months after stroke (26). The SIS is an eight-domain self-report questionnaire that is used to assess muscle strength, memory, emotion, communication, ADL, mobility, hand function, and participation. Additionally, the SIS includes an additional question in which participants are asked to score their global perceived stroke recovery on a vertical visual analog scale (VAS) ranging from 0 to 100, with 0 representing no recovery and 100 representing complete recovery (26). The SIS VAS score was used to assess stroke recovery in this study. A difference of 10 in the VAS score measured at two different time points was considered clinically meaningful (27).

The Hospital Anxiety and Depression Scale (HADS) subscale, the HADS-A, was used to assess anxiety (28). The HADS is a self-report instrument considered useful for assessing clinically significant anxiety and depression symptoms. The HADS-A consists of seven items, each rated on a 4-point scale, with a total score of 21. Anxiety symptoms with a score of ≥8 are considered clinically significant (28).

The NIHSS was used to assess stroke-related neurological impairment (29). The total NIHSS score was 42 (range 0–42), with 0 indicating no neurological deficit, and NIHSS scores ≤3 were considered mild stroke (30). The NIHSS is a reliable instrument for assessing stroke severity in an emergency setting (29).

Performance in basic ADL was assessed using the BI, which is an ordinal scale consisting of 10 items. The BI scale ranges from 0 to 100, with higher scores indicating greater independence (31). For example, scores ranging from 61 to 90 indicate moderate ADL dependence, 91–99 indicate slight ADL dependence, and 100 points indicate complete ADL independence (32).

The Montreal Cognitive Assessment (MoCA) was used to assess cognitive function. The total MoCA score ranges from 0 to 30, with lower scores indicating cognitive impairment (33). A score of ≥26 indicates normal cognitive function (34).

Other variables analyzed in this study were sex, age, and type of stroke defined according to the International Classification of Diseases 11th revision criteria (I61, non-traumatic intracerebral hemorrhage,; I63, cerebral infarction) (35). In addition, we collected data on stroke localization using the medical charts of the patients.

### Statistical analysis

Descriptive statistics were used to describe the characteristics of the study sample (mean [Standard deviation] and median [interquartile range, and min-max]). Many variables hade skew distributions, therefore, non parametric statistical testes were used. The Mann–Whitney U-test was used for continuous variables (age, NIHSS, MoCA and BI) and Chi-square test for categorical variables (sex) were used to compare the included and excluded participants.

### Cross-sectional analysis between perceived stroke recovery and anxiety

**Figure 2:**
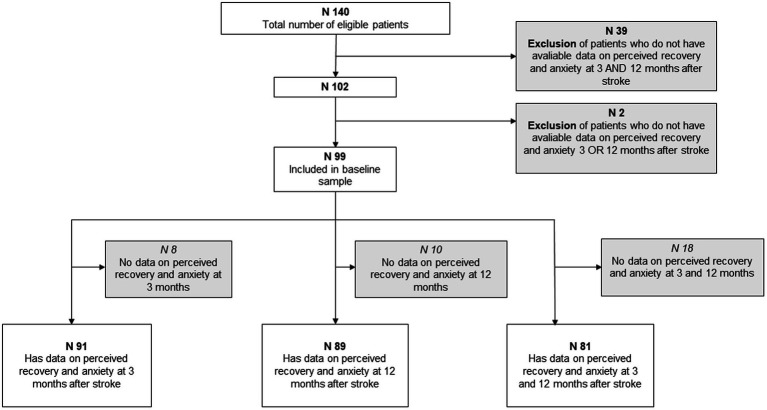
Flow chart summarizing the inclusion and exclusion criteria of the baseline sample and three group divisions.

To decrease the proportion of missing data three different selection for analyses was done depending on available data. One group included all participants who had data on stroke recovery and anxiety at 3 months after stroke (*n* = 91), one group who had data on both variables at 12 months after stroke (*n* = 89), and one group that had available data on both variables at 3 and 12 months (81) (Figure 2). In other words each participant could be included in several groups.

At 3 and 12 months, the groups were classified into an anxiety group (HADS-A ≥ 8) and a no-anxiety group (HADS-A < 8). The Mann–Whitney U test was used to detect any differences in stroke recovery between the anxiety and no-anxiety groups at 3- and 12-months after stroke. The non-parametric Spearman’s rank-order correlation was used to investigate the relationship between stroke recovery and anxiety at 3 and 12 months. Correlation values were classified as small (*r*_s_ < ±0.29), medium (*r*_s_ = ±0.30 to ±0.49), and large (*r*_s_ ≥ ±0.50) (36). Shared variance (%) was calculated using *r*_s_^2^ × 100, where *r*_s_ is the Spearman’s rank-order correlation coefficient.

Participants with data on anxiety and stroke recovery at 3- and 12-months after stroke were included in the longitudinal analyses. The Wilcoxon signed-rank test was used to investigate whether anxiety and stroke recovery significantly changed from 3 to 12 months.

Data were dichotomized based on the anxiety scores of the participants in the anxiety group (HADS-A ≥ 8, at both 3 and 12 months) and no-anxiety group (HADS <8 at 3 and/or 12 months). To express stroke recovery as a continuous and categorical variable, the change in stroke recovery from 3 to 12 months was calculated, and the results were trichotomized according to clinically significant positive change (+10 points or more), clinically significant negative change (−10 points or less), or no change (±9). The Mann–Whitney U test was used to analyze if there were differences in the change in stroke recovery between the anxiety and no-anxiety groups.

The Mann–Whitney U test was also used to compare clinically significant negative and positive changes in stroke recovery with the no-change group concerning anxiety scores at 3 and 12 months after stroke.

Statistical analyses were performed using SPSS Statistics software (IBM SPSS Statistics for Windows, Version 27.0.; IMB corp., Armonk, NY, United States) (37). The significance level for all statistical tests was set at α = 5%.

## Results

Based on data on anxiety and stroke recovery at 3 and/or 12 months, 99 of the 140 participants met the inclusion criteria. The 99 participants were further divided into three groups, depending on the available data at the time. The groups consisted of 91, 89 and 81 participants, respectively, (Figure 2). The included participants were not statistically different from the excluded participants in terms of age (*p* = 0.58) and stroke severity assessed using the NIHSS (*p* = 0.86). However, the exclusion group had significantly more male participants than females compared to the included group (*p* = 0.03).

Of the 99 participants, 44.4% were female, and the median age at the onset of stroke was 77 years. The median stroke severity measured using the NIHSS 2 days after the stroke was 1.0 (range, 0–11), and the majority of participants (89%) had a mild stroke (NIHSS ≤ 3) (Table 1).

**Table 1:** Baseline characteristics of the study sample, *n* = 99.

Baseline characteristics	*N* (%)	Mean (SD)	Median (IQR [min-max])
Age (years)		74.4 (12.0)	77 (15 [36–96])
Length of hospital stay in days		13 (5.8)	11 (8 [1–37])
Stroke severity 2 d after stroke (NIHSS)		1.6 (2.12)	1.0 (2 [0–11])
Mild stroke (NIHSS ≤3)	65 (89)		
Stroke type			
Ischemic	93 (93.9)		
Hemorrhagic	6 (6.1)		
Stroke localization			
Right	29 (29.3)		
Left	23 (23.2)		
Bilateral	4 (4.0)		
Cerebellum	8 (8.1)		
Brainstem	2 (2.0)		
Unknown	33 (33.3)		
Sex			
Male	55 (55.6)		
Female	44 (44.4)		
Activities of daily living (BI)		78.1 (15.89)	80 (25 [50–100])
Severe - moderate dependence (BI <90)	66 (67.3)		
Slight dependence (BI 90–99)	21 (21.4)		
Independent (BI = 100)	11 (11.1)		
Cognitive function (MoCA)		21.7 (4.7)	22 (7 [10–29])
Normal cognitive function (MoCA ≥26)	19 (25.3)		
Impaired cognitive function (MoCA <26)	56 (74.7)		

Variables with missing data, *n* (%): NIHSS 26 (26.3); BI 1 (1.01); MoCA 24 (24.2). SD, standard deviation; IQR, interquartile range; NIHSS, National Institutes of Health Stroke Scale; BI, Barthel Index, MoCA, Montreal Cognitive Assessment.

### Perceived stroke recovery and anxiety 3 months after stroke

Three months after the stroke (*n* = 91), the median scores for stroke recovery and anxiety were 80 and 3 points, respectively (Table 2). Sixteen (17.6%) of the participants were classified into the anxiety group (HADS-A ≥ 8). Participants who experienced anxiety 3 months after stroke had significant higher NIHSS scores 2 days after stroke (*p* = 0.02). However, there was no significant difference in age, sex or BI between those with anxiety and those without. Participants in the anxiety group had a significantly lower median stroke recovery rate than participants in the no-anxiety group (*p* < 0.001).

**Table 2:** Change of anxiety and stroke recovery from 3- to 12-months.

	3 Months (*n* = 91)	12 Months (*n* = 89)	*p*
**Anxiety**			
Median (IQR [min-max])	3.0 (0–18)	3 (0–16)	*0.78*
Mean (SD)	3.9 (4.3)	3.7 (3.6)	
Possible occurrence of anxiety, *n* (%)	16 (17.6%)	14 (15.7%)	
**Stroke recovery**			
Median (IQR [min-max])	80 (25–100)	80 (0–100)	*0.19*
Mean (SD)	74.3 (21.1)	77.3 (19.9)	

SD, standard deviation; IQR, interquartile range. *n* = sample size.

**Figure 3:**
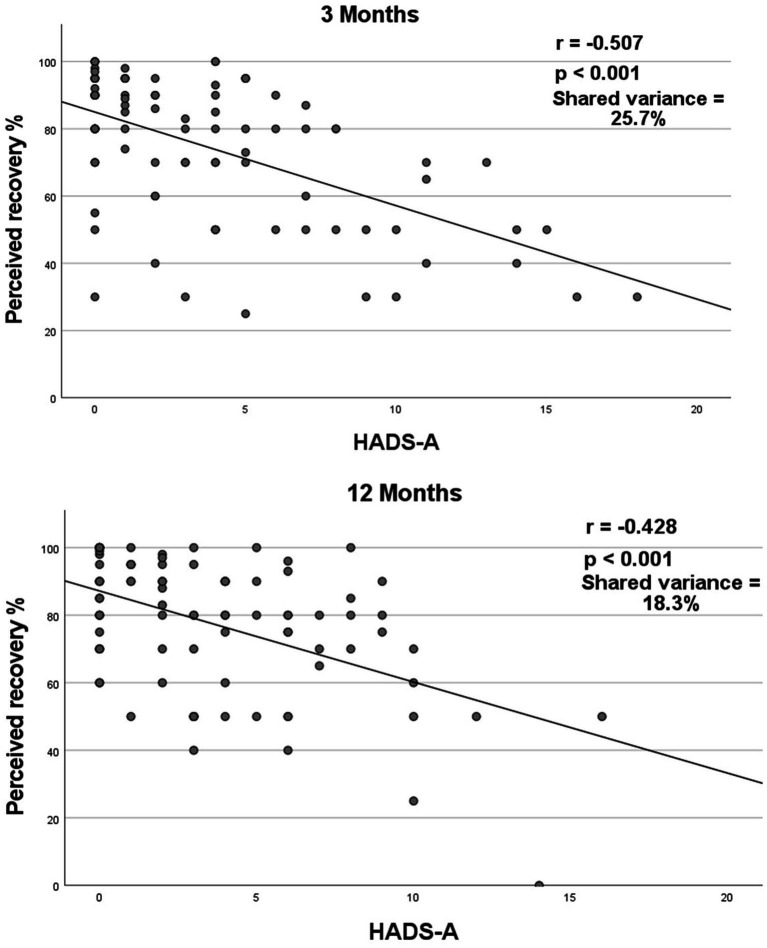
Correlation between stroke recovery and anxiety 3- and 12-months post-stroke. Spearman’s correlation coefficient (*r*_s_) between stroke recovery and HADS-A at 3- and 12-months post-stroke. HADS-A = Hospital Anxiety and Depression Scale subscale of anxiety. Correlation value interpretation: small (*r*_s_ < ±0.29), medium (*r*_s_ = ±0.30 to ±0.49), and large (*r*_s_ = ±0.50 to ±1.0) (27).

Anxiety and stroke recovery were found to have a large negative correlation (*r*_s_ = −0.507, *p* < 0.001; Figure 3). A high level of anxiety was associated with a lower level of stroke recovery 3 months after the stroke. The shared variance between anxiety and stroke recovery was 25.7%, indicating that 25% of the patients who exhibited anxiety symptoms showed a lower rate of stroke recovery.

### Perceived stroke recovery and anxiety 12 months after stroke

Twelve months after the stroke (*n* = 89), the median scores for stroke recovery (VAS score of SIS) and anxiety (HADS-A) were 80 and 3 points, respectively, (Table 2). Fourteen (15.7%) of the participants were classified into the anxiety group (HADS-A ≥ 8), and had a significantly lower median recovery rate than participants in the no-anxiety group (*p* = 0.018). Twelve months after stroke, no statistical difference was detected between patients with anxiety and those without, with respect to age, gender, NIHSS scores or BI scores.

Anxiety and stroke recovery were found to have a moderately negative correlation (*r*_s_ = −0.428, *p* < 0.001; Figure 3). A high level of anxiety was associated with a lower level of stroke recovery 12 months after the stroke. The shared variance between anxiety and stroke recovery was 18.3%, indicating that 18.3% of the patients who exhibited anxiety symptoms showed a lower rate of stroke recovery.

### Longitudinal changes in perceived stroke recovery and anxiety from 3 to 12 months

Eighty-one participants had data on anxiety and stroke recovery at 3- and 12-months after stroke. No significant change from 3- to 12-months was observed in stroke recovery (*p* = 0.19) or anxiety (*p* = 0.78; Table 2).

From 3 to 12 months, 17 (21%) participants had a clinically meaningful positive stroke recovery, 46 (56.8%) had no clinically meaningful change in their stroke recovery, and the remaining 18 (22.2%) participants had a stroke recovery change that was negative.

The anxiety group included seven (8.6%) participants with HADS-A ≥ 8 at both time points. Compared to the no-anxiety group, the anxiety group showed no significant difference in the change in stroke recovery from 3- to 12-months (*p* = 0.67; Table 3).

**Table 3:** Baseline characteristics depending on anxiety or no anxiety at 3- and 12-months post stroke.

	3 Months		*p*	12 Months		*p*
	Anxiety *n* = 16	No anxiety *n* = 77		Anxiety *n* = 14	No anxiety *n* = 76	
Age mean (SD)	75.7 (12.5)	73.9 (12.3)	0.41	76.8 (8.8)	73.6 (12.7)	0.45
Sex M/F	8/8	43/34	0.78	6/8	45/31	0.38
NIHSS (day 2) mean (SD)		1.4 (1.8)	0.02	1.9 (2.1)	1.5 (2.2)	0.52
BI mean (SD)	75 (12.8)	78.4 (16.6)	0.31	73.6 (14.2)	78.3 (16.3)	0.27
HADS (day 5) mean (SD)	9.2 (3.8)	3.9(3.6)	<0.001	8.3 (3.7)	3.7 (3.7)	<0.001

## Discussion

The findings revealed that participants with anxiety at both time points did not experience clinically significant changes in stroke recovery. However, the subgroup of participants who experienced anxiety at 3 or 12 months reported more positive recovery. In addition, a significant association was found between anxiety and lower levels of stroke recovery at 3- and 12-months after stroke. Based on these results, we could not establish a causal relationship between anxiety and stroke recovery. Therefore, more attention should be paid to anxiety after a stroke, as it may have implications for stroke recovery.

At 3- and 12-months after stroke, participants with anxiety had a lower stroke recovery rate than those without anxiety. A previous study found a significant association between post-stroke anxiety and decreased quality of life (7). Although our study did not assess quality of life, it can be assumed that the degree of perceived stroke recovery is associated with quality of life, which to some extent is consistent with our findings. Some studies have also found an association between anxiety and lower self-perceived ADL (16), while others found no association (10, 38). The use of different stroke outcome assessment tools could explain the disparity with other studies. We chose SIS as an outcome measure in our study to emphasize the personal perceived stroke recovery of the participants, which includes their expectations, needs, and values. The relationship between anxiety and stroke recovery remains unclear. Anxiety can affect the rehabilitation process and delay stroke recovery because it affects the mood, motivation, sleep, and compliance of the patients. However, those who experience slower recovery may develop anxiety because of a lack of improvement. Since stroke recovery is a multidimensional process, it can also be affected by factors other than anxiety, such as stroke localization and severity, age, comorbidities, motivation, social support, and others (39).

There was no difference in stroke recovery from 3 to 12 months after stroke between participants with and without anxiety. This could be due to the low prevalence of anxiety among our participants, which was 16 and 18% at 3- and 12-months, respectively. Eight percent of the participants reported anxiety at both time points and could be analyzed as an anxiety group in longitudinal analyses. Our findings for the longitudinal anxiety group differ from those of other studies, which found a 20–30% prevalence of anxiety after stroke (9, 10). One explanation for this disparity could be our classification criteria for the anxiety group, which required participants to have anxiety at both assessment time points (3- and 12-months after stroke). This may have resulted in a lower prevalence of anxiety. In addition, because our study mostly consisted of patients with mild to moderate stroke, few participants may have experienced anxiety and thus had a lower impact on daily life. Anxiety was assessed in our study using the HADS-A scale, which has been shown to be a useful tool for assessing anxiety symptoms (28). Other studies have used various tools to screen anxiety and subgroup participants in the anxiety group; however, the HADS-A has been the most commonly used tool (10). The Diagnostic and Statistical Manual of Mental Disorders, Fifth Edition is another well-known tool used to diagnose anxiety. Mental health professionals use it to diagnose and classify mental disorders based on specific criteria (40). It encompasses various anxiety disorders, including generalized anxiety disorder, post-traumatic stress disorder, and others (41). The HADS-A is used to screen for anxiety symptoms but cannot diagnose anxiety, whereas The Diagnostic and Statistical Manual of Mental Disorders, Fifth Edition can be used by a professional to diagnose a specific disorder. Therefore, the actual number of participants with anxiety disorders may differ, leading to different findings.

We found no significant change in stroke recovery from 3- to 12-months. This finding contradicts that of another study that found a significant improvement in perceived stroke recovery during the same period (20). Another study found no significant difference in stroke recovery between patients with mild stroke, whereas those with more severe strokes experienced a significant change (42). This is consistent with our findings since most of our participants had mild strokes and, thus, experienced no significant change in stroke recovery. Anxiety levels did not significantly change from 3- to 12-months after stroke. This is consistent with another study, in which anxiety showed no significant change over the same time (12) and could be explained, as mentioned before, by a lower prevalence of anxiety, as our study mostly consisted of mild strokes.

In this study, participants who had anxiety at the 3- or 12-month follow-up showed a greater clinically significant positive change than those with no anxiety. This implies that participants who experienced anxiety at 3- or 12-months had a higher change in positive stroke recovery than those with no anxiety. One possible reason could be that those with higher anxiety levels 3 months after a stroke had a significantly lower stroke recovery and therefore had more room for improvement. Furthermore, another reason could be that participants who had expressed their recovery as low at 3 months due to any impairment caused by the stroke were later able to adjust to their lives, and expressed their stroke recovery as significantly higher at 12 months than at 3 months. Others may have been less frustrated with their lives 3 months after the stroke; nevertheless, there was less to adjust to, leading to no significant change in their stroke recovery 9 months later. Age, sex or BI at baseline did not differ between those with anxiety or not at the 3- nor at the 12-months follow up. However, there was a significant difference in NIHSS and HADS-A at baseline between those with anxiety or not at the 3-months follow up, were those with anxiety had higher NIHSS and higher HADS-A. A possible reason for this can be that if you have a higher NIHSS score you are likely to have more symptoms to be concerned about and therefore perhaps rate higher on the HADS-A. At the 12-months follow up the only significant difference at baseline between those with anxiety or not was the HADS-A, were those with anxiety estimated higher on the HADS-A at baseline. It is not unreasonable to assume that those with anxiety at 3- and 12-months follow up also had anxiety at baseline.

The participants in this study were analyzed as a whole cohort because the original GOTVED study found no difference between the intervention and control groups (24). To the best of our knowledge, this is the first study to explore the relationship between anxiety and perceived stroke recovery. The SIS proved to be a reliable tool with which we were able to assess the relationship between anxiety and perceived stroke recovery, as we were interested in assessing the stroke recovery perception of each patient. We also considered the self-awareness of each participant for their stroke recovery by using the SIS, as self-awareness can be affected by stroke. However, other health issues that participants may have encountered during the longitudinal analysis can influence the self-assessment of stroke recovery, thereby influencing recovery assessment. Our data were obtained from the stroke unit of the largest hospital network in Sweden. Therefore, the population from which the participants in this study were enrolled was diverse. One assessor performed follow-up assessments, and the participants completed the self-assessment using the relevant tools under the supervision of the assessor. One limitation is that most of our participants experienced mild strokes because we used data from a randomized controlled trial of the GOTVED study, which had strict inclusion and exclusion criteria. Therefore, this study may not have represented the entire population, specifically those who experienced severe strokes. This is a limit which affects the generalizability of our results. However the GOTVED study did not show any significant difference between the groups regarding the primary outcome, anxiety, and therefore we consider that the data can be used as a cohort. In addition it has been shown that the characteristics of the stroke population has changed, showing a strong shift towards mild strokes (43), which suggests that our results are generalizable. Anxiety was assessed using the HADS-A scale, a reliable tool for assessing anxiety symptoms. As a cutoff point, we chose ≥8 points for participants to have anxiety, which is consistent with other studies (9). We, unfortunately have no data on previous history of psychologic disorders.

Our findings indicate a connection between anxiety and stroke recovery; however, further research with constructive data collection is necessary to increase the generalizability of the findings.

## Conclusion

Anxiety after a stroke is common and is associated with lower levels of perceived stroke recovery for at least 1 year after the stroke. To identify patients at risk of low recovery, it may be beneficial to identify those with anxiety early after stroke and offer psychological treatment as early as possible. Anxiety management may improve stroke recovery and quality of life in patients.

## Data availability statement

The raw data supporting the conclusions of this article will be made available by the authors, without undue reservation.

## Ethics statement

The studies involving humans were approved by Swedish Ethical Review Authority (formerly Regional Ethical Review Board) in Gothenburg. The studies were conducted in accordance with the local legislation and institutional requirements. The participants provided their written informed consent to participate in this study. Written informed consent was obtained from the individual(s) for the publication of any potentially identifiable images or data included in this article.

## Author contributions

LN: Writing – original draft. LR: Writing – review & editing. TA: Writing – review & editing. KS: Writing – review & editing.

## Abbreviations

HADS-A: Hospital Anxiety and Depression Scale – Subscale of Anxiety
SIS: Stroke Impact Scale
VAS: Visual Analog Scale
GOTVED: Gothenburg Very Early Supported Discharge study
ADL: activities of daily living
NIHSS: National Institutes of Health Stroke Scale
BI: Barthel Index
MoCA: Montreal Cognitive Assessment
